# Muscle mitochondrial capacity in high‐ and low‐fitness females using near‐infrared spectroscopy

**DOI:** 10.14814/phy2.14838

**Published:** 2021-05-15

**Authors:** Bart Lagerwaard, Joëlle J. E. Janssen, Iris Cuijpers, Jaap Keijer, Vincent C. J. de Boer, Arie G. Nieuwenhuizen

**Affiliations:** ^1^ Human and Animal Physiology Wageningen University and Research Wageningen The Netherlands; ^2^ TI Food and Nutrition Wageningen The Netherlands

**Keywords:** fitness, mitochondria, NIRS, oxidative metabolism, $\dot{V}$O_2_peak

## Abstract

The recovery of muscle oxygen consumption (m$\dot{V}$O_2_) after exercise measured using near‐infrared spectroscopy (NIRS) provides a measure of skeletal muscle mitochondrial capacity. Nevertheless, due to sex differences in factors that can influence scattering and thus penetration depth of the NIRS signal in the tissue, e.g., subcutaneous adipose tissue thickness and intramuscular myoglobin and hemoglobin, it is unknown whether results in males can be extrapolated to a female population. Therefore, the aim of this study was to measure skeletal muscle mitochondrial capacity in females at different levels of aerobic fitness to test whether NIRS can measure relevant differences in mitochondrial capacity. Mitochondrial capacity was analyzed in the *gastrocnemius* muscle and the wrist flexors of 32 young female adults, equally divided in relatively high ($\dot{V}$O_2_peak ≥ 47 ml/kg/min) and relatively low aerobic fitness group ($\dot{V}$O_2_peak ≤ 37 ml/kg/min). m$\dot{V}$O_2_ recovery was significantly faster in the high‐ compared to the low‐fitness group in the *gastrocnemius*, but not in the wrist flexors (*p* = 0.009 and *p* = 0.0528, respectively). Furthermore, $\dot{V}$O_2_peak was significantly correlated to m$\dot{V}$O_2_ recovery in both *gastrocnemius* (*R* ^2^ = 0.27, *p* = 0.0051) and wrist flexors (*R* ^2^ = 0.13, *p* = 0.0393). In conclusion, NIRS measurements can be used to assess differences in mitochondrial capacity within a female population and is correlated to $\dot{V}$O_2_peak. This further supports NIRS assessment of muscle mitochondrial capacity providing additional evidence for NIRS as a promising approach to monitor mitochondrial capacity, also in an exclusively female population.

## 1 INTRODUCTION

Regular endurance exercise increases whole‐body peak oxygen uptake ($\dot{V}$O_2_peak) due to bodily adaptations that increase oxygen transport, delivery and consumption. At the level of the skeletal muscle, maximal oxygen consumption increases due to an increase in muscle mitochondrial mass and function (Lanza & Nair, 2009). The exact contribution of this increased skeletal muscle oxidative capacity to the improved $\dot{V}$O_2_peak after regular endurance exercise remains debated. Nevertheless, there appears to be a strong link between mitochondrial mass and $\dot{V}$O_2_peak (Weibel et al., 1991). Furthermore, skeletal muscle oxidative capacity is suggested to be a determining factor in prolonged strenuous exercise performance (Holloszy & Coyle, 1984). Classically, skeletal muscle oxidative or mitochondrial capacity is analyzed ex vivo, by measuring oxygen consumption in permeabilized muscle fibers from muscle biopsies. The invasive nature of this technique, the isolation of the tissue from its physiological environment, as well as the infringement of cell integrity by the permeabilization procedure provides a rationale for non‐invasive assessment of muscle mitochondrial capacity in an intact system.

A near‐infrared spectroscopy (NIRS)‐based technique has been developed to assess skeletal muscle mitochondrial capacity in vivo (Nagasawa et al., 2003). Using multiple, transient vascular occlusions after a short bout of exercise it allows for the measurement of post‐exercise recovery of m$\dot{V}$O_2_ (Motobe et al., 2004). The underlying assumption is that post‐exercise regeneration of readily available energy carriers, i.e., ATP and phosphocreatine (PCr), is directly linked to aerobic metabolism and, therefore, a higher mitochondrial capacity will be associated with a faster recovery to the pre‐exercise state (McMahon & Jenkins, 2002). NIRS offers advantages over other non‐invasive techniques, such as magnetic resonance spectroscopy (^31^P‐MRS), due to its higher portability and relatively low‐costs, making it more suitable for on‐site and routine measurements. However, a limitation of the NIRS technique is the limited penetration depth in the tissue, as the greater the distance the NIR light has to travel to reach muscle tissue, the lower the resolution (van Beekvelt et al., 2001). Therefore, factors that influence the scattering of the signal, such as differences in subcutaneous adipose tissue thickness (ATT), but also skin thickness, skin pigmentation and blood flow (van Beekvelt et al., 2001; Craig et al., 2017; Wassenaar & Van den Brand, 2005) can affect the NIRS measurement of post‐exercise recovery of m$\dot{V}$O_2_.

In a normally active male population, we previously showed that NIRS is able to detect differences in mitochondrial capacity in the *gastrocnemius* muscle between relatively high‐ and low‐fitness subjects, and this NIRS‐derived measure of mitochondrial capacity was correlated to $\dot{V}$O_2_peak (Lagerwaard et al., 2019). Nevertheless, it is unsure if these results are easily extrapolated to a female population, because of the aforementioned factors that could affect the signal to noise ratio, such as ATT thickness, which can be anticipated to be larger in females, and total hemoglobin and myoglobin, which was shown to be lower in females in the *gastrocnemius* muscle, but not in other muscles, e.g., wrist flexors, compared to males (Craig et al., 2017). Additionally, it could be that possible sex differences in the relationship between mitochondrial capacity $\dot{V}$O_2_peak also affect this relationship. For instance, males showed a larger stimulation of mitochondrial biogenesis than females upon sprint interval training (Scalzo et al., 2014). Indeed, straightforward extrapolation is warned, as a recent NIRS study showed no correlation between mitochondrial capacity in the *gastrocnemius* muscle and $\dot{V}$O_2_peak when males and females were combined (Beever et al., 2020), which contrasted our previous findings in males only (Lagerwaard et al., 2019).

Thus, even though studies in mixed population indicate the application of the technique in both sexes (Brizendine et al., 2013; Hamaoka et al., 2011; Ryan, Brizendine et al., 2013; Ryan et al., 2014; Sako et al., 2001), it is unknown whether NIRS is able to detect physiologically relevant differences within an exclusively female population, a population also often underrepresented in sports and exercise research (Costello et al., 2014). Therefore, the aim of this study is to measure skeletal muscle mitochondrial capacity in healthy females at different levels of aerobic fitness to further support the applicability of NIRS assessment of mitochondrial capacity in this population. Mitochondrial capacity was measured in both the frequently activated *gastrocnemius* muscle and the often‐undertrained wrist flexors in 32 recreationally active, healthy females divided into a relatively low and a relatively high‐fitness group. We hypothesized that high‐fitness females will show a higher mitochondrial capacity compared to low‐fitness females in both muscles.

## 2 MATERIALS AND METHODS

### 2.1 Ethical approval

The study was approved by the medical ethical committee of Wageningen University with reference number NL70136.081.19. All procedures performed were in accordance with the ethical standards of the institutional and/or national research committee and with the 1964 Helsinki declaration and its later amendments or comparable ethical standards (Fortaleza, Brazil 2013). The study is registered in the Dutch trial register (NL7891). Written informed consent was obtained from all individual participants included in the study.

### 2.2 Subjects

Healthy females between the age of 18–28 years were recruited from the local university and community population. None of the subjects had a history of cardiovascular, respiratory or metabolic disease. None of the subjects were a regular smoker (>5 cigarettes per week), used recreational drugs during the study or reported recent use of performance enhancing drugs or supplements. Subjects were non‐anemic (hemoglobin concentration >12 g/dl), verified by using HemoCue Hb 201 microcuvette (HemoCue AB). None of the subjects were pregnant or lactating. 17β‐estradiol levels were measured with at Erasmus Medical Centre, The Netherlands using chemiluminescent immunoassay on Lumipulse G1200 analyzer (Fujirebio Inc). Subjects that used any other monophasic oral contraceptive containing low synthetic estradiol and progesterone were excluded from participation. Test days were planned within the end of the follicular phase until menstruation, based on self‐reported occurrence of last menstruation or during final 14 days of pill cycle.

### 2.3 Pre‐experimental screening protocol

Subjects were selected based on $\dot{V}$O_2_peak, measured using an incremental exercise test on electrically braked bicycle ergometer (Corival CPET, Lode). Subjects were asked to refrain themselves from vigorous exercise for 48 h and to have consumed their last meal two hours before this test. Oxygen consumption, carbon dioxide production and air flow were measured using MAX‐II metabolic cart (AEI technologies). Exhaled air was continuously sampled from a mixing chamber and heart rate was measured with a strap‐on chest heart rate monitor (Polar Electro). After a 3‐minute unloaded cycling warming‐up, the protocol started at a workload of 50 W for subjects who exercised <3 times a week or 75 W for subjects who exercised >3 times per week and was increased every minute in increments of 15 W. Subjects were instructed to maintain a self‐selected pedal rate between 70–80 revolutions per minute (RPM). Inability to pedal at a rate above 60 RPM for 10 s was considered point of exhaustion and the end of the test. For the test to be valid, two out of three of the following criteria should have been met: (1) a maximal heart rate within 10 beats of the predicted maximum (220–age), (2) attainment of a plateau in $\dot{V}$O_2_, i.e. $\dot{V}$O_2_ failing to increase with 150 ml/min, despite an increase in workload, and (3) achievement of an RER ≥1.1. The $\dot{V}$O_2_peak was determined by binning data in 15 s intervals. 16 relatively high‐fitness ($\dot{V}$O_2_peak ≥47 ml/kg/min) and 16 low‐fitness subjects ($\dot{V}$O_2_peak ≤ 37 ml/kg/min) were selected to take part in the study, based on chosen cutoffs. Main exercise modalities in the high‐fitness group were running/athletics (6×), rowing (3×), kickboxing (2×), hockey (1×), swimming (1×), ice skating (1×), climbing (1×), and weightlifting (1×). Main exercise modalities in low‐fitness group were aerobics (2×), horseback riding (1×), weightlifting (1×), climbing (1×), walking (1×), dancing (1×), badminton (1×), or no regular exercise (8×). A total of 111 exercise tests were conducted to end up with the desired sample size.

### 2.4 Experimental protocol

The subjects refrained from heavy physical exercise 48 h prior to testing and from any exercise and consumption of alcohol 24 h prior to testing. Maximal Voluntary Contraction (MVC) hand grip strength of the non‐dominant and dominant hand was measured using a Jamar Hydraulic Hand Dynamometer (Performance Health). Highest value out of three 5 s isometric contractions was set as MVC. Body fat percentage was measured according to the four‐site method by Durnin–Womersley using the skinfold measurements of the triceps, biceps, sub scapula and supra iliac, measured using a skinfold caliper. Furthermore, skinfold between NIRS receiver and transmitter was measured on the calf and the forearm.

### 2.5 NIRS measurements

Deoxyhemoglobin (HHb) and oxyhemoglobin (O_2_Hb) were continuously measured using the continuous wave Oxymon, dual‐wavelength NIRS system (760 and 850 nm; Artinis Medical Systems) at three optode distances 15, 45 and 55 mm. Data were collected via Bluetooth at 10 Hz using the Oxysoft software (Artinis Medical Systems). The NIRS probe was placed longitudinally on the belly of the muscle, identified by palpation by an experienced investigator, on the medial *gastrocnemius* and on the wrist flexors of the non‐dominant side. To secure the probe and protect it from environmental light, the probe was tightly taped to the skin. To measure oxygen consumption, a blood pressure cuff (Hokanson SC5 and SC12; D.E. Hokanson Inc.) was placed proximally of the probe above the knee joint and on the upper arm. The cuff was powered and controlled by a rapid cuff inflator system (Hokanson E20 and AG101 Air source; D.E. Hokanson Inc.) set to a pressure of 230–250 mm Hg. Post‐exercise muscle oxygen consumption recovery was assessed similar to previously published protocols (Ryan, Southern, et al., 2013). In summary, the protocol consists of three 30 s rest measurements of resting oxygen consumption. To calibrate the signal between individuals, the minimal‐oxygenation (0%) of the tissue underneath the probe was determined by 30 s maximal hand grip exercise for wrist flexors or by plantar flexion exercise using a rubber resistance band for *gastrocnemius*, followed by a 4‐min arterial occlusion. The hyperemic response after the cuff was released was considered maximal oxygenation (100%). Recovery of muscle oxygen consumption after exercise was measured after 30 s of intermittent (approximately 0.5 Hz) handgrip exercise at 40% of MVC for the wrist flexors or plantar flexion exercise using a rubber resistance band until 50% of maximal oxygenation for *gastrocnemius*. Right after exercise, a series of transient occlusions (5*5 s on/5 s off, 5*7 s on/7 s off, 10*10 s on/10 s off) was used to measure the recovery of muscle oxygen consumption after exercise. Recovery measurements were performed in duplicate with 2 min rest in between tests.

### 2.6 Analysis of muscle oxygen consumption data

NIRS data were analyzed using Matlab‐based (The Mathworks) analysis software (NIRS_UGA). Optode distance of 45 or 55 mm was used, based on inspection of data of raw light counts during measurements. Data were analyzed as % of maximal oxygenation. m$\dot{V}$O_2_ was calculated during every arterial occlusion using the slope of the change in HHb and O_2_Hb (Hb difference) for 3 s for the 5 s occlusions, for 5 s for the 7 s occlusions, 7 s for the 10 s occlusions and 15 s for the basal measurements. A blood volume correction factor was used for each data point (Ryan et al., 2012) to correct for redistribution of blood distally from the cuff. In short; changes in HHb and O_2_Hb should be proportional during arterial occlusions. A blood volume correction factor (β) was calculated to account for possible changes and was used to correct each data point. m$\dot{V}$O_2_ recovery measurements post‐exercise were fitted to a mono‐exponential curve:

$$y\;\left(t\right)=End‐\;\Delta *e^{‐k\;∙t}$$

with y representing the m$\dot{V}$O_2_ during the arterial occlusions; End being the m$\dot{V}$O_2_ immediately after the cessation of exercise; delta (∆) being the difference between m$\dot{V}$O_2_ after exercise and m$\dot{V}$O_2_ during rest; k being the rate constant expressed in time units; t being time. Recovery of muscle oxygen consumption follows mono‐exponential curve (Meyer, 1988), therefore data points outside curve were considered artifacts and omitted from curve fitting. Data were analyzed blinded by two researchers. In case of discrepancy between analyses, third researcher analyzed data set (blinded) and consensus was reached. Rate constants calculated from curve fitting with *R* ^2^ < 0.95 were excluded from analysis as a measure of poor data quality. Rate constants of duplicates were averaged.

### 2.7 Statistical analyses

Data are presented as mean ± SD, unless indicated otherwise. Statistical analyses were performed using GraphPad Prism v.5 (GraphPad Software). Means between the two groups were compared using a Students unpaired t‐test. Correlations between variables were compared using regression analysis. Significance was accepted at *p* < 0.05. Normality was tested using Shapiro‐Wilk normality test. Not‐normal data were compared using Mann‐Whitney tests.

## 3 RESULTS

Physical characteristics are shown in Table 1. All subjects completed all tests without any contraindications. All maximal exercise tests met at least two out of three preset criteria. Subjects were either on monophasic oral contraceptive containing synthetic estradiol and progesterone (N = 13), used a copper spiral (N = 1) or did not use any contraceptives (N = 18). The use of oral control contraceptives was N = 7 in high‐fitness and N = 6 in low‐fitness group. No significant difference in 17β‐estradiol levels was observed between the high‐fitness and low‐fitness group in subjects that did not use hormonal birth control contraceptives (Table 1).

**TABLE 1:** Subjects' characteristics

	Low‐fitness (n = 16)	High‐fitness (n = 16)
Age (years)	24.0 [21.3–25.5]	21.8 [21.5–23.6]
Ethnicity	Caucasian (11), Asian (1), Indo‐pacific (4)	All Caucasian
Weight (kg)	59.2 ± 7.2	60.8 ± 6.9
Height (m)	1.63 ± 0.07	1.68 ± 0.04*
Fat mass (% of weight)	28.9 ± 3.9	24.6 ± 4.7**
$\dot{V}$O_2_peak (ml/kg/min)	35.1 [32.2–35.7]	51.0 [49.2–55.4]***
MVC dominant arm	30.0 [25.3–33.5]	36.5 [32.0–39.50]*
MVC non‐dominant arm	27.5 [24.0–33.5]	33.5 [30.3–37.0]*
Hemoglobin (mmol/L)	8.4 ± 0.6	8.5 ± 0.6
Usage of birth control pill	6/16	7/16
If not;17β‐estradiol (pmol/L)	470.9 [337–590]	153.8 [84−806]
ATT wrist flexors (mm)	5.3 [4.3–6.9]	4.0 [2.3–5.0]*
ATT GAS (mm)	8.6 [6.9–10.6]	6.9 [6.0–7.9]*

Maximal oxygen consumption ($\dot{V}$O_2_peak), maximal voluntary contraction (MVC) handgrip strength, adipose tissue thickness (ATT), gastrocnemius (GAS). Values are mean ± SD for normally distributed data, and median [Inter quartile range] for not normally distributed data.

* *p *< 0.05.

** *p* < 0.01.

*** *p *< 0.001.

### 3.1 Recovery of m$\dot{V}$O_2_ in gastrocnemius and wrist flexors

**FIGURE 1:**
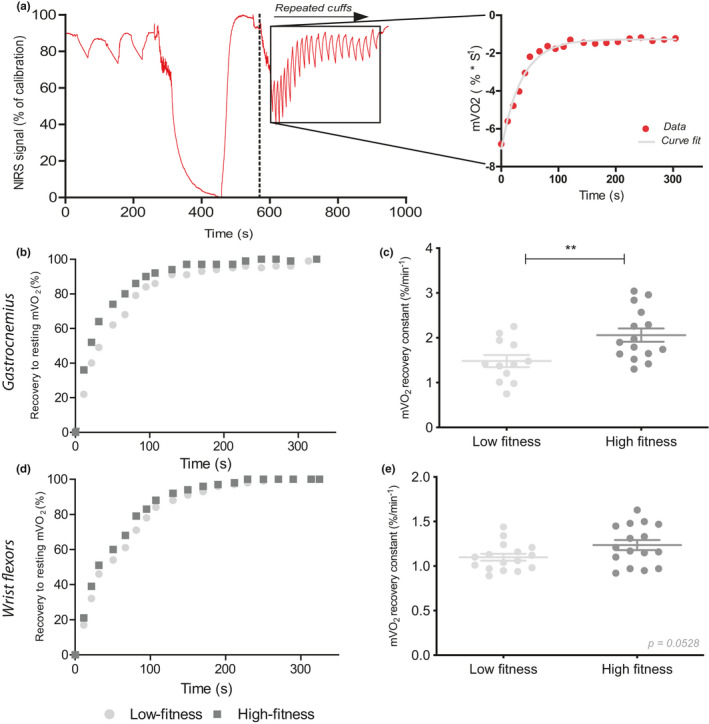
Representative plot of NIRS protocol. Red line represents NIRS signal of the Hb difference during protocol as percentage of maximal oxygenation. Repeated measurement m$\dot{V}$O_2_ (red dots) are fitted to a monoexponential curve fit (grey line) from which a recovery constant is derived (a). Average curve fits for the low‐fitness and high‐fitness group for m$\dot{V}$O_2_ recovery presented as percentage of basal m$\dot{V}$O_2_ after 30 s of plantar flexion exercise in *gastrocnemius* (b) and after a handgrip exercise in wrist flexors (d). Recovery constants derived from monoexponential curve fits for *gastrocnemius* (c) and wrist flexors (e). For *gastrocnemius* muscle n = 12 versus n = 15. Values are represented as mean ± SD. **p < 0.01

The NIRS protocol, which was used both for the *gastrocnemius* and wrist flexors, included 3 measurements of basal m$\dot{V}$O_2_, assessment of minimal and maximal oxygenation level and the repeated occlusions to assess recovery of oxygen consumption after a short exercise protocol (Figure 1a). For *gastrocnemius*, two data sets were excluded due to *R* ^2^ < 0.95, two were excluded due to failed calibration measurement, i.e. no plateau for minimal oxygenation was reached, and one data set was excluded due to technical issues (only had 15 mm channel). For all other measurements, plateau for minimal oxygenation was reached. Recovery rate constants were significantly different between the high‐ and low‐fitness group for *gastrocnemius* (2.06 ± 0.57 vs. 1.48 ± 0.47, *p* = 0.009; Figure 1b,c), but not for the wrist flexors (1.24 ± 0.23 vs. 1.10 ± 0.15, *p* = 0.0528; Figure 1d,e).

### 3.2 Relationship between m$\dot{V}$O_2_ recovery and whole‐body oxygen uptake

**FIGURE 2:**
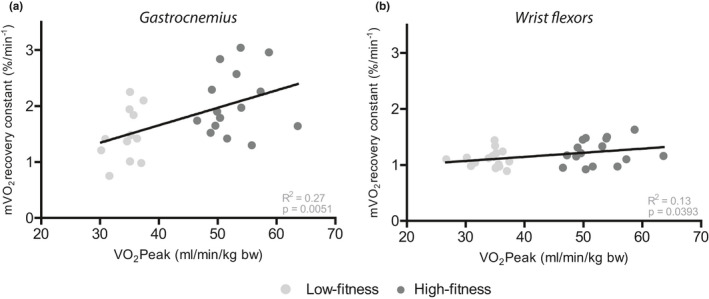
Correlation between maximal oxygen consumption ($\dot{V}$O_2_Peak) measured during an incremental exercise test and recovery constants for muscle oxygen consumption recovery (m$\dot{V}$O_2_) measured using NIRS in *gastrocnemius* (a) calculated after 30 s of plantar flexion and wrist flexors (b) calculated after 30 s of handgrip exercise at 50% of MVC in the high‐fitness (dark grey) and low‐fitness (light grey) group

In order to test the relationship between endurance capacity, measured as $\dot{V}$O_2_peak, and m$\dot{V}$O_2_ recovery, measured using NIRS, a correlation analysis was performed. The m$\dot{V}$O_2_ recovery constant of the *gastrocnemius* was significantly correlated to $\dot{V}$O_2_peak (Figure 2a; *R* ^2^ = 0.27, *p* = 0.0051). Furthermore, in the wrist flexors a significant correlation was observed between m$\dot{V}$O_2_ recovery constant and $\dot{V}$O_2_peak (Figure 2b; *R* ^2^ = 0.13, *p* = 0.0393).

## 4 DISCUSSION

The aim of this study was to measure skeletal muscle mitochondrial capacity using NIRS in healthy females in the *gastrocnemius* and wrist flexors muscles to test whether NIRS can measure relevant differences in mitochondrial capacity and to further support NIRS assessment of mitochondrial capacity in this population. We are the first to show that recovery of m$\dot{V}$O_2_ after a short bout of exercise as measure for mitochondrial capacity is significantly faster in the *gastrocnemius* muscle of high‐fitness compared to low‐fitness individuals in an exclusively female population. Recovery of m$\dot{V}$O_2_ in the wrist flexors muscle was not statistically different in between two groups. Furthermore, when taking both groups together, we found a significant correlation between $\dot{V}$O_2_peak and recovery of m$\dot{V}$O_2_ in the *gastrocnemius* and wrist flexors.

### 4.1 m$\dot{V}$O_2_ recovery in gastrocnemius between high‐ and low‐fitness females

This study shows that NIRS is able to detect physiological relevant differences in mitochondrial capacity in a healthy, recreationally active female population. The differences in mitochondrial capacity likely reflect a higher mitochondrial capacity in high‐fitness individuals, i.e., more or more efficient mitochondria were able to reinstate muscle homeostasis faster. In a previous, but unique, study we showed a 40% faster m$\dot{V}$O_2_ recovery in the *gastrocnemius* muscle of high‐fitness compared to low‐fitness males (Lagerwaard et al., 2019). The difference in magnitude of $\dot{V}$O_2_peak was comparable with the current study, in which we likewise observed a 40% faster m$\dot{V}$O_2_ recovery in high‐fitness compared to low‐fitness females. Brizendine et al. showed an approximate doubling of mitochondrial capacity in the *vastus lateralis* muscle in endurance athletes compared to inactive individuals (Brizendine et al., 2013). Although this study included both males and females, the vast majority of the endurance athletes were males and with an absolute difference in $\dot{V}$O_2_peak of 40 ml/kg/min between the groups, the distinction between the groups was twice as large compared to the current study. Therefore, the present study highlights the sensitivity of NIRS measurements of m$\dot{V}$O_2_ recovery to detect smaller differences in mitochondrial capacity and further extends the applicability of the technique, also in an exclusively female population.

The highly comparable results between in m$\dot{V}$O_2_ recovery in the *gastrocnemius* muscle between the two sexes indicates the applicability of this NIRS‐based technique to detect physiological relevant differences also in an exclusively female population. This is an important finding, as sex differences that could affect the light scattering, and thus the m$\dot{V}$O_2_ recovery measurement, have been identified, such as a lower total hemoglobin and myoglobin in the *gastrocnemius* (Craig et al., 2017) and generally higher ATT in females compared to males. Higher levels of ATT can greatly affect the NIR‐signal and consequently the signal to noise ratio of the measurement (van Beekvelt et al., 2001; Craig et al., 2017). Even though in the current experimental protocol the difference in ATT was accounted for by normalization of the signal within each person using a physiological calibration (Hamaoka et al., 1996). Still, interrogation depth of the muscle is decreased with increasing ATT and this can result in a substantial attenuation of the signal from muscle tissue, such that a doubling of ATT from 4 to 8 mm reduces the contribution of total‐[Hb+Mb] to the signal by 50% using a 20 mm source‐detector distance (Craig et al., 2017). In the current population, average ATT on the *gastrocnemius* muscle was 8.1 mm, which was expectedly higher than previously observed in males (5.9 mm) (Lagerwaard et al., 2019). Not many studies have measured in these ranges of ATT in females (Adami et al., 2017; van Beekvelt et al., 2001; Beever et al., 2020; Brizendine et al., 2013; Southern et al., 2014). Yet, studies that did measure close to our range in ATT either reported difficulties (Adami et al., 2017), adapted the penetration depth according to the ATT per individual (Brizendine et al., 2013; Ryan, Southern, et al., 2013) or used a frequency‐domain NIRS device that can better quantify the degree of light scattering (Ryan et al., 2014).

To overcome the relatively high ATT, we used a greater source‐detector distance of 45 or 55 mm in females, compared to 35 mm in males. A greater source‐detector distances allows for deeper tissue penetration and consequently increased attribution of muscle to the NIR‐signal. Nevertheless, increasing source‐detector distance will also cause less light to reach the detector, as more signal is lost due scattering in the tissue. Still, our results showed that with distances of 45 and 55 mm, tissue penetration and signal to noise ratios were sufficiently high to obtain reliable, i.e. *R* ^2^ > 0.95, m$\dot{V}$O_2_ recovery curves and to were able to identify differences in m$\dot{V}$O_2_ recovery between two fitness groups in the *gastrocnemius* muscle a healthy, recreationally active female population. Nevertheless, two data sets were excluded from analysis due to low curve fitting, or *R* ^2^. The data sets excluded for low *R* ^2^ were among the highest in ATT thickness (10.95 and 11.05 mm). Therefore, although other measurements with higher ATT (e.g., 11.05 and 11.35 mm) were successful, and the NIR signal is also affected by other factors such as optode placement, exercise execution and movement artifacts, it could be that the larger contribution of adipose tissue to the NIR signal negatively affected the reliability of the m$\dot{V}$O_2_ recovery curves. Therefore, our results suggest that increasing the source‐detector distance is an effective, yet limited, approach for the application of NIRS to assess mitochondrial capacity in muscles with a substantial ATT.

All test days were planned within the end of the follicular phase until menstruation, i.e. luteal phase, based on self‐reported occurrence of last menstruation. Due to variation of estradiol levels in luteal phase and the effect of 17β‐estradiol on mitochondrial capacity (Torres et al., 2018), differences in 17β‐estradiol levels could have affected the m$\dot{V}$O_2_ recovery measurements. Yet, we observed no difference in 17β‐estradiol between the two groups and we did not observe a correlation between 17β‐estradiol and m$\dot{V}$O_2_ recovery (data not shown). For these reasons, it is unlikely that differences in circulating levels 17β‐estradiol could explain the significant difference in m$\dot{V}$O_2_ recovery between the two groups. Nevertheless, we cannot rule out effects of the menstrual cyclical patterns, as, for example, neuromuscular function and fatigability showed modulations based of the phase of menstrual cycle in knee extensor muscles (Ansdell et al., 2019). Yet, because the current study also included monophasic oral contraceptive users, effects of menstrual cycle on the outcome measures could expected to be limited.

### 4.2 The relationship between aerobic fitness and m$\dot{V}$O_2_ recovery

Although our primary aim was to find differences in m$\dot{V}$O_2_ recovery in high‐ and low‐fitness females, when taking both groups together, we found a significant correlation between $\dot{V}$O_2_peak and recovery of m$\dot{V}$O_2_ in the *gastrocnemius*. A correlation between mitochondrial capacity of skeletal muscle tissue and $\dot{V}$O_2_peak has been established before. For instance, several NIRS studies describe a correlation between m$\dot{V}$O_2_recovery and $\dot{V}$O_2_peak, in particular in the *gastrocnemius* muscle of males (Lagerwaard et al., 2019), and in the *vastus lateralis* of mixed populations (Beever et al., 2020; Brizendine et al., 2013). Such a correlation has also been observed in a female population, using ^31^P‐MRS, showing the rate of PCr resynthesis in the *gastrocnemius* muscle was correlated to $\dot{V}$O_2_peak (Larson‐Meyer et al., 2000). Comparable to NIRS, ^31^P‐MRS uses the recovery of muscle homeostasis after exercise, assessed by measuring the regeneration of PCr as a proxy for mitochondrial capacity (Meyer, 1988; Nagasawa et al., 2003) and the two techniques show a good agreement (Ryan, Southern, et al., 2013; Sako et al., 2001). Even though the correlations in the current study should be treated with caution because of the discontinuous distribution of the $\dot{V}$O_2_peak values, our data are in agreement with the established correlation between skeletal muscle mitochondrial capacity and $\dot{V}$O_2_peak, further supporting the applicability and physiological relevance of this technique in females.

### 4.3 m$\dot{V}$O_2_ recovery in wrist flexors between high‐ and low‐fitness females

Although a significant difference was found in m$\dot{V}$O_2_ recovery in the *gastrocnemius* muscle, a significant difference in m$\dot{V}$O_2_ recovery was not observed in wrist flexors between high‐fitness and low‐fitness females. This result is similar to data obtained in males with similar differences in $\dot{V}$O_2_peak (Lagerwaard et al., 2019). However, with a p‐value near significance and the weak correlation between m$\dot{V}$O_2_ recovery and $\dot{V}$O_2_peak, one might argue that a slight increase in sample size would have resulted in a statistically significant difference. Nevertheless, not considering statistical significance, the difference m$\dot{V}$O_2_ recovery kinetics is rather small and could be less biologically relevant. This discrepancy between the wrist flexor and *gastrocnemius* muscle might be attributed to less frequent activation of the wrist flexors during endurance exercise and consequently less mitochondrial adaptations, such as increased amount and the efficiency of the mitochondria (Hamner et al., 2010). Therefore, although the wrist flexors are a convenient muscle group to measure due to low ATT levels and exercise standardization, it is likely a poorer reflection of aerobic fitness. Therefore, m$\dot{V}$O_2_ recovery kinetics in this muscle should therefore not be used as a predictor for aerobic capacity or exercise performance.

### 4.4 Conclusion and further perspectives

This study provides evidence for sensitive measurements of mitochondrial capacity using NIRS in a female population. In a population of healthy, recreationally active females, mitochondrial capacity was significantly higher in the *gastrocnemius* of high‐fitness compared to low‐fitness females. Furthermore, mitochondrial capacity was significantly correlated to $\dot{V}$O_2_peak. These results further substantiate the use of m$\dot{V}$O_2_ recovery as a measure for mitochondrial capacity measured non‐invasively using NIRS as a relevant physiological parameter. Furthermore, these results support the applicability of this technique to detect relevant physiological differences in a female population with higher ATT by using a physiological calibration and greater source‐detector distances. However, increasing source‐detector distance comes with limitations, such as decreased signal intensity at the detector due to the scattering of light in the tissue. Furthermore, one has to consider portability, as commercially available portable NIRS often have a smaller maximal source‐detector distance compared to wired NIRS optodes (Perrey & Ferrari, 2018). Portability, besides relative faster testing and lower costs, is a promising feature of the NIRS assessment of mitochondrial capacity, allowing measurements in an onsite, field‐based setting. Therefore, testing different populations should be considered good practice to further increase applicability of the technique.

Additionally, in a recent study looking at predictors of exercise performance on a time‐to‐completion cycling trial, it was shown that m$\dot{V}$O_2_ recovery measured using NIRS best predicted performance on the trial (Batterson et al., 2020). This supports the physiological relevance of NIRS assessment of m$\dot{V}$O_2_ recovery assessment as a relevant marker in sports and exercise science. Additionally, NIRS has advantages over established techniques, such as less invasive than a muscle biopsy and more portable and lower cost compared to ^31^P‐MRS. Moreover, recently it has been shown that using a 6‐occlusion protocol is a valid and reproducible alternative to protocols using more occlusions, such as the current one (Sumner et al., 2020). Using a shorter protocol reduces testing time or could, if desired, increase replicates to increase precision of the measurement. The strong prediction for exercise performance, relative fast testing and the portability, appoint m$\dot{V}$O_2_ recovery measurements using NIRS as a promising approach to monitor aerobic performance in both laboratory and field‐based settings, also in females.

## CONFLICT OF INTEREST

No conflicts of interest, financial or otherwise, are declared by the authors. The Companies FrieslandCampina and Danone Nutricia Research are sponsors of the TIFN program and partly financed the project. They had no role in data collection and analysis, decision to publish, or preparation of the manuscript, but commented the study design.

## AUTHOR CONTRIBUTION

BL, JJEJ, IC performed all experiments. BL, IC performed principal data analysis; BL, JJEJ, AGN, VCJB., JK conception and design of research; data analysis and interpretation. BL drafting of manuscript. All authors edited, revised and approved final version of manuscript.
